# Electricity as Applied to Chemical and Metallurgical Processes

**Published:** 1901-10

**Authors:** J. Conard Watkins

**Affiliations:** Winston-Salem, N. C.


### International Dental Journal.

Vol. XXII. October, 1901. No. 10.

### Original Communications.[1]

### ELECTRICITY AS APPLIED TO CHEMICAL AND METALLURGICAL PROCESSES.[2]

BY J. CONARD WATKINS, D.D.S., WINSTON-SALEM, N. C.

Mr. President and Members of the North Carolina State Dental Society,—Chemistry and metallurgy of to-day are very little like they were a few years ago. We look with ridicule and pity at the efforts of the alchemists as they tried to turn mercury into gold, forgetting that our own chemists wrought infinitely greater magic when they discovered the process whereby at will they could extract from foul, filthy coal-tar all the colors of the rainbow and all the sweet perfumes of Arabia. Chemistry of to-day has gone forth into the highways and hedges of industry and commerce working magic all along its course. There is no art and no manufacture, however insignificant, that has not come under its beneficent influence to a greater or less extent.

One of the most important factors in bringing about these great improvements is the marvellous development of electricity. We are now familiar with the telegraph, the telephone, the electric

[1] The editor and publishers are not responsible for the views of authors of papers published in this department, nor for any claim to novelty, or otherwise, that may be made by them. No papers will be received for this department that have appeared in any other journal published in the country.

[2] Read before the North Carolina Dental Association, June 26 to 28, 1901.

railway and automobile, the X-ray apparatus, the electric light, the new system of wireless telegraphy, and many other kinds of electrical appliances.

We think it would not be inappropriate to speak of electricity as applied to chemical and metallurgical processes. In doing this, we will mention a few of these practical changes which are likely to prove of interest as well as of great importance in our profession.

Most of the very little that has actually been accomplished in electro-chemistry has been done by use of electrolysis and electro-synthesis. We will describe the preparation of one of our important dental medicines that is prepared by these methods, which will give us a practical idea of the *modus operandi.*

### IODOFORM.

Ethyl alcohol in an alkaline iodide solution by the aid of electrolysis gives us iodoform, which is represented in the following equation:

$$CH_3CH_2OH + 10I + H_2O = CHI_3 + CO_2 + 7HI$$

This electrolysis is best performed with ten to fifteen grammes calcined soda and ten grammes potassium iodide in one hundred cubic centimetres of water and twenty cubic centimetres of alcohol, which are placed in a porous earthen cylinder with a platinum anode. The cathode, of nickel, is surrounded by a strong solution of sodium hydroxide. A temperature of 70° C. is required with a current density at the anode of one ampere per one hundred square centimetres, and is continued for from two to four hours. After standing for several hours iodoform crystallizes from the solution. Sodium iodide remains in the mother liquor as the principal secondary product.

### ALUMINUM.

Thirty-eight years ago aluminum was a chemical curiosity. Through the efforts of Deville it first acquired a commercial character, and its extraction became a metallurgical process. But now the development of electrometallurgy has enabled us to produce aluminum economically.

In 1889 the aluminum produced in this country was mainly obtained from Greenland cryolite. The Pittsburg Reduction Company in this country carries on the extraction of aluminum from its oxide, alumina, by means of electrolysis. The principle is that the alumina is decomposed in the presence of a melted fluoride by the electric current, and metallic aluminum is liberated.

The furnace used in the extraction of aluminum is an open, iron-cased box, which is thickly lined with carbon and is provided with a spout at the bottom for "tapping off" the aluminum. The alumina is dissolved in the "fused flux," consisting of fluorides of aluminum and sodium, which serves as a vehicle for the alumina. A large block, or series of bars, of carbon, carried on an adjustable support and arranged to dip into the centre of the furnace, forms the anode, the furnace itself forming the cathode. After the flux and alumina are introduced, the carbon anode is brought well down into the furnace and the current is turned on. Considerable resistance is at first offered, but as the materials in the furnace become highly heated this decreases, and the anode can be raised slightly.

Decomposition soon begins, and the oxygen is liberated at the anode, where it unites with carbon, forming carbon dioxide, which passes off, while the aluminum remains. The metallic aluminum is heavier than the melted bath, and as it sinks to the bottom it is "tapped off" from time to time, while fresh alumina is added.

The uses of aluminum are varied and without end. It is used for drinking-utensils, smokers' sets, watch-cases, umbrella-handles, billiard-cues, and an endless variety of souvenirs.

Then, again, we have often seen references in the newspapers to the Swiss steam-launch of aluminum. As early as 1891 an aluminum life-boat was constructed in Prussia. In the German army aluminum canteens and military equipments are used. We are also familiar with the many dental uses of aluminum, the more important of which are for impression-trays, air-chambers, aluminum combination dentures and plates. In operative dentistry we meet with aluminum crowns and bridges.

Aluminum is a splendid conductor of electricity and is now largely used in electrical appliances. The hardness of aluminum varies according to its purity, the purest being the softest. Like gold, it hardens on being worked, and annealing makes it very soft. In hardness aluminum stands next to gold.

In ductility aluminum ranks sixth among the metals. Aluminum is very malleable, standing second only to gold. Aluminum can be rolled into sheets of only from five to seven ten-thousandths of an inch in thickness, and such sheets can be beaten into leaf nearly as thin as any gold-leaf. This aluminum-leaf is largely used in decorative work, and is it not possible that it can, some day, be substituted for gold-foil in operative dentistry?

For a long time it was thought that aluminum could not be soldered, and for this reason its practical use was somewhat retarded. We know that chloride of silver will act as a solder for aluminum, and the Pittsburg Reduction Company claims that there are preparations that will nicely solder aluminum. If this be true, may we not predict that in the near future we will see a great many aluminum dentures?

### CARBORUNDUM.

Ten years ago Mr. Edward G. Acheson, of Monongahela City, Pa., in the face of many obstacles, carried to commercial success his invention of carborundum. Within these few years carborundum has rapidly risen, until it stands in the front rank of abrasives.

Many of us in the preparation of teeth for crown- and bridge-work, as well as in prosthetic dentistry, use carborundum with a great degree of satisfaction, for we can with it cut much faster than with the other abrasives.

At first there was some hesitation on the part of the profession in adopting carborundum. Among other objections was the fact that it was very expensive; but now, the time saved and the lessening of "torture" to the patient (and those who have had live teeth prepared for a crown know that it *is* torture) give it a fixed place in dentistry.

This new material stands next to diamond, not only in composition, but in its internal and external properties. Its crystals are wonderfully brilliant, and its extreme hardness makes it an efficient and powerful abrasive material.

This wonderful product is obtained by heating a mixture of one hundred parts sand, twenty-five parts salt, and twenty-five parts coke in an electric furnace. It has been found that the addition of sawdust to the mixture renders it porous, which allows the gas to escape. The output of the first furnaces was one-quarter of a pound of carborundum a day; now a single furnace yield is about seven thousand pounds of crystalline carborundum.

From time to time improvements in the furnaces have been made, until to-day the works at Niagara Falls under the direction of Mr. Acheson have gigantic furnaces, of which we will speak later.

We now turn our attention to the manufacture of carborundum. The crude materials are received in the stock building, and, with the exception of the coke, are ready for immediate use.

The coke is passed through a grinder and carried to the top of the building, where it is poured through successive screens, from which the powder is passed on for the mixture, while the kernels of a certain size are landed into the core-bin and are used in making the core.

The sand, coke, sawdust, and salt are conveniently conveyed into the scales, from which they are elevated to the mechanical mixer. All the work of preparing this mixture is so systematized and mechanically done that it requires the labor of *only two* men.

The furnaces, of which there are fourteen, are built of brick in the form of an oblong box, the approximate interior dimensions being sixteen feet in length, five feet in width, and five feet high. The ends of the furnace are the only permanent part, as the remainder is built up each time the furnace is used. The ends are about two feet thick, and through their centres pass the terminals, which consist of sixty carbon rods thirty inches long and three inches in diameter. "The outer ends of the carbons are enclosed in a square iron frame, to which is screwed a stout plate bored with sixty holes, corresponding to the ends of the carbons. Through each of these holes is passed a short piece of three-eighths-inch copper rod, fitting tightly a hole drilled in the carbon.

"Finally all the carbons are tightly packed with graphite. Each plate is provided with four projections, to which the cables conveying the current may be bolted."

We now look at the furnace proper. The side-walls are built up about four feet. Pieces of sheet-iron are so placed as to keep the mixture four inches from the inner ends of the carbon terminals. The furnace is then filled about half full of the mixture. Between the ends a semicircular trench with a radius of about ten and one-half inches is made into which the proper amount of the carbon kernels is placed, and they are rounded with the hand to form a cylinder twenty-one inches in diameter.

Between the terminals and sheet-irons finely ground coke is packed and the sheet-irons are then removed, which completes the connection. The walls of the furnace are built up about five feet in height, and the mixture is heaped on until it rises to about eight feet, when you are ready for the electric current.

The Niagara Falls Power Company furnishes power that has an electro-motive force of two thousand two hundred volts, but as this is too high for use in the furnace, it is reduced in a transformer that has a maximum capacity of eleven hundred horse-power, and reduces the two thousand two hundred volt current into one of only one hundred and eighty-five volts. The circuit is completed and is regulated by a volt-metre and an am-metre. Of course, the resistance of the core is very great at first, but after a while it becomes sufficiently low to allow one thousand horse-power to be steadily employed.

After the circuit has been on about half an hour we notice the peculiar odor of escaping gas. After three or four hours the sides and top of the furnace are completely enveloped by a lambent blue flame, which is that of carbon monoxide. At the end of about thirty-six hours the current is cut off and the furnace allowed to cool for a few hours. After taking down the side walls and removing the unchanged mixture we reach the outer crust of amorphous carborundum. This is easily removed with large steel bars, and can readily be separated from the inner crust of amorphous carborundum, which is removed with a spade, and we now find *carborundum crystals.* The carborundum is then taken to the crusher, where large rollers break apart the mass of crystals. These crystals are then taken to large wooden tanks, where for several days they are treated with dilute sulphuric acid to remove impurities. They are then thoroughly washed, dried, and passed through screens which grade them from No. 8 to No. 220.

Care is used that "nothing be lost," and the water that washes the crystals is passed through a series of tanks, where the fine powder, "flowers," is obtained. This is graded F, FF, and FFF.

The vitrified process is used for forming the carborundum crystals into wheels. The carborundum is mixed with a certain proportion of kaolin and feldspar, and after the mixture is placed into a mould hydraulic pressure is applied.

The vitrification of the wheels is carried out in kilns similar to those used in baking porcelain. The operation of firing lasts about seven days, after which the wheels are "turned up," tested, and then they are ready for the market.

Carborundum costs from two to five times as much as emery, but in tests with emery it has been proved that the same results can be obtained at less expense with carborundum than with emery.

The uses of carborundum are varied, and we will only mention a few. Carborundum is used in the manufacture of steel as a substitute for ferro-silicon; it is also used by the watchmaker, the roll-grinder, the pearl-grinder, and, in fact, wherever an abrasive is required.

Already the carborundum companies are prepared to furnish eighty thousand different wheels, not including dental wheels and other specialties.

Before concluding we must mention some of the leading characteristics of carborundum. In hardness it is between nine and ten degrees,—*nearer* ten,—which is the hardness of diamond. It cuts emery or corundum with ease. It is not as tough as diamond, but is brittle, closely resembling corundum. It is infusible in the highest attainable heat. Carborundum is insoluble in all the ordinary solvents. Acids, oils, water, and not even hydrofluoric acid will dissolve it.

It is composed of silicon and carbon in equal atomic proportions, and its chemical formula is $SiC$.

The color of pure carborundum is white. In commerce we meet many colors and shades, the prevailing colors being blue, green, and black; these are partly the result of impurities and partly owing to the surface oxidation, but this has no effect whatever upon its hardness.

Chemistry and metallurgy by the means of electricity during the last ten years have brought us so many new and important products, that after a thoughtful consideration of the remarkable achievements of the past century it might seem to the laity that the limit of scientific research had almost been reached. But the thinking man knows that this work is not done; in fact, it is but commenced. There is an infinity of problems *yet* to be solved, and let *us* await with eager anticipation, ever ready to accept what the twentieth century may unfold.

